# A critical evaluation of *Mycobacterium bovis* pangenomics, with reference to its utility in outbreak investigation

**DOI:** 10.1099/mgen.0.000839

**Published:** 2022-06-28

**Authors:** Kristina M. Ceres, Michael J Stanhope, Yrjö T. Gröhn

**Affiliations:** ^1^​ Department of Population Medicine and Diagnostic Sciences, College of Veterinary Medicine, Cornell University, Ithaca, New York, USA; ^2^​ Population and Ecosystem Health, College of Veterinary Medicine, Cornell University, Ithaca, NY, USA

**Keywords:** *Mycobacterium tuberculosis *complex, pangenomics, molecular epidemiology

## Abstract

The increased accessibility of next generation sequencing has allowed enough genomes from a given bacterial species to be sequenced to describe the distribution of genes in the pangenome, without limiting analyses to genes present in reference strains. Although some taxa have thousands of whole genome sequences available on public databases, most genomes were sequenced with short read technology, resulting in incomplete assemblies. Studying pangenomes could lead to important insights into adaptation, pathogenicity, or molecular epidemiology, however given the known information loss inherent in analyzing contig-level assemblies, these inferences may be biased or inaccurate. In this study we describe the pangenome of a clonally evolving pathogen, *Mycobacterium bovis *, and examine the utility of gene content variation in *M. bovis* outbreak investigation. We constructed the *M. bovis* pangenome using 1463 *de novo* assembled genomes. We tested the assumption of strict clonal evolution by studying evidence of recombination in core genes and analyzing the distribution of accessory genes among core monophyletic groups. To determine if gene content variation could be utilized in outbreak investigation, we carefully examined accessory genes detected in a well described *M. bovis* outbreak in Minnesota. We found significant errors in accessory gene classification. After accounting for these errors, we show that *M. bovis* has a much smaller accessory genome than previously described and provide evidence supporting ongoing clonal evolution and a closed pangenome, with little gene content variation generated over outbreaks. We also identified frameshift mutations in multiple genes, including a mutation in *glpK*, which has recently been associated with antibiotic tolerance in *Mycobacterium tuberculosis *. A pangenomic approach enables a more comprehensive analysis of genome dynamics than is possible with reference-based approaches; however, without critical evaluation of accessory gene content, inferences of transmission patterns employing these loci could be misguided.

## Data Summary

Sequence reads are available on the National Centre for Biotechnology Information (NCBI) Sequence Reads Archive. A full list of accession numbers for sequences used in this study are available in Supplemental File 1. The *M. bovis* gene presence/absence matrix is available in Supplemental File 2. The quality of genome assemblies was assessed using contig count, N50 and checkM completeness and contamination measures in addition to manual screening. Data processing scripts are publicly available on https://github.com/kmceres/Mbovis_pangenome. Supplementary materials are available on Figshare: https://doi.org/10.6084/m9.figshare.19398161 [1].

**Impact Statement**

Dissecting true gene content variation, from noise generated by analysis of incomplete genomes, is a necessary step to understand the role of horizontal gene transfer in the evolution of the *M. bovis* pangenome. Here we show that analysis of incomplete genomes can lead to incorrect identification of accessory genes, which artificially inflates the pangenome size. When correcting for these annotation errors, we show that *M. bovis* evolves clonally, and has a very compact accessory genome. We also show that there is limited variation in gene content among outbreak sequences and that this variation is driven by indels, suggesting indel variation could be useful in distinguishing between highly similar SNP profiles in epidemiological analysis.

## Introduction

*Mycobacterium bovis* is a member of the *Mycobacterium tuberculosis* complex (MTBC), the collection of pathogenic bacteria including *M. tuberculosis *, which causes tuberculosis in humans and *M. bovis *, which causes bovine tuberculosis in cattle and other mammalian species. MTBC species are unique among bacterial taxa because unlike many species, they have highly conserved genomes and are thought to evolve strictly clonally [2, 3]. A consequence of strict clonality is relatively little gene content variation compared to species that can generate diversity through horizontal gene transfer (HGT).

In addition to HGT, variability in gene content can arise through *de novo* gene formation, or through gene deletion. The competing forces of gene gain and gene loss create a U-shaped gene frequency spectrum, with many genes shared by most genomes on one end of a gene frequency spectrum, and a similar number of rare genes on the other end. Since MTBC genomes are thought to evolve clonally, without HGT, MTBC accessory genomes are hypothesized to be very small, and have fewer rare genes compared to other taxa. Recent MTBC studies, however, challenge the idea of a small accessory MTBC genome, with estimates of MTBC accessory genome size ranging from 2086 genes (36 % of all genes) [4] to 7620 genes (69 % of all genes) [5]. Importantly, a recent *M. bovis* study by Reis and Cunha suggests that *M. bovis* has an open pangenome [6], implying new *M. bovis* genes would be discovered with each new genome sequenced. If *M. bovis* does have an open pangenome, it would suggest *M. bovis* does not evolve clonally.

Similarly, studies investigating homologous recombination in MTBC have yielded conflicting results. Experimental evidence has shown that while the closely related free-living *Mycobacterium canettii* could accept donor DNA via homologous recombination, MTBC could not [2]. However, despite this lack of experimental evidence, computational methods sometimes identify recombination in MTBC genomes [4, 7, 8]. Some of the recombination in MTBC species identified through computational methods stems from poor quality data or unreliable alignments. For example, Godfroid *et al*. identified an imbalance in the number of recombination events between terminal branches and internal nodes, with a skew towards more recombination events on terminal branches and an unequal distribution of recombination events among genomes [9]. Furthermore, phylogenies created with these erroneous regions conflicted with the established *M. tuberculosis* phylogenies, leading the authors to conclude that any true recombination signal that was identified was greatly outweighed by noise stemming from poor data quality [9].

Resolving the conflict between bioinformatic detection of recombination and large accessory genomes and experimental evidence suggesting clonality is necessary to understand MTBC genome dynamics and their implication for outbreak analysis and the evolution of important phenotypes like antimicrobial resistance. Whole genome sequencing is an indispensable tool in outbreak investigation, enabling the identification of epidemiological links between individuals or groups that would otherwise be difficult to ascertain by comparing pathogen sequences. However, accurate reconstruction of a phylogenetic tree relies on identifying regions of the genome that evolve clonally because phylogenetic trees are usually bifurcating trees where each node has exactly one parent. To accurately model evolution of a pathogen with proportions of the sequence evolving through HGT, nodes must be allowed to have more than one parent reflecting the inheritance of genetic material from more than one parental source, leading to a network structure or ancestral recombination graph, rather than a bifurcating tree structure. Therefore, to infer transmission relationships using a bifurcating tree, regions of the genome that are affected by recombination are often excised from alignments. If there is ongoing HGT in *M. bovis* and other MTBC species, alignments used to create trees relevant to transmission analysis must be carefully curated to ensure only clonally evolving genome segments are included. Additionally, if MTBC species have true accessory genomes gene content variation could be used in outbreak analysis to discriminate between highly similar core genome sequences.

Here we investigate the evolutionary forces that shape the *M. bovis* pangenome and explore evolutionary scenarios that could have resulted in observed gene content variation using *de novo* assembled draft *M. bovis *. We look for evidence of homologous recombination and true gene content variation, and carefully examine accessory genes for assembly or annotation errors. Since there is no known mechanism for HGT in the MTBC, we suspect that gene deletion and gene duplication are the major drivers of pangenomic diversity in the MTBC. Consequently, after accounting for technical errors created during sequencing or assembly or bioinformatic misclassification, we expect that gene content variation patterns will closely resemble core genome single nucleotide polymorphism (SNP) variation patterns. We show that *M. bovis* has a much smaller accessory genome than previously described and provide evidence supporting ongoing clonal evolution. This finding suggests careful examination of pangenome and recombination results from bioinformatic analyses is necessary to accurately describe MTBC genome dynamics.

## Methods

### Sequence assembly and annotation

Raw *M. bovis* sequence files from Almaw *et al*. [10], Loiseau *et al*. [11], Orloski *et al*. [12], Reis and Cunha [7], Rodrigues *et al*. [13], Zwyer *et al*. [14] and from BioProject PRJNA769553 were downloaded from the NCBI Sequence Read Archive. A full list of downloaded samples can be found in Supplemental File 1. Sequences were assembled using SPAdes genome assembler v3.14.0 [15] in careful mode, and polished using Pilon v1.23 [16]. The quality of assemblies was assessed using QUAST v5.0.2 [17] and checkM v1.1.3 [18]. To minimize annotation issues inherent with draft genomes, only sequences with less than or equal to 150 contigs and less than 5 % contamination were used for further analysis. It was noted upon visual inspection that some contigs contained duplicate gene sequences that were the reverse compliment of each other, such that the entire contig was one palindromic sequence. Our view is this was likely caused by assembly error, and therefore systematically identified all such palindromic sequences (81 contigs total) by searching for contigs that contained reverse compliments of the same gene with identical DNA sequences. Each identified contig was visually inspected, and sequences that had any confirmed palindromes were removed. Sequences were annotated using prokka v1.14.16 [19] using the AF2122/97 (NCBI accession NC_002945.4) *M. bovis* reference genome as a guide [20].

### Sequence selection

Before assembling genomes, we determined which lineage each sequence belonged to according to the new *M. bovis* lineage classification scheme suggested by Zwyer *et al*. [14]. We called SNPs for all downloaded fastq using the vSNP pipeline (https://github.com/USDA-VS/vSNP) against the *Mycobacterium tuberculosis* H37Rv reference genome (NCBI accession NC_000962.3). SNPs were filtered out if they had a read depth less than 10, an allele frequency less than 0.9, and a mapping quality less than 30 using vcffilter in vcflib v1.0.2 [21]. Lineages were assigned using H37Rv genome positions in Table 4 of Zwyer *et al*. [14].

### Pangenome analysis

Panaroo was used to characterize the *M. bovis* pangenome [22]. To identify the reference coordinates of partial genes undergoing pseudogenization, the consensus gene sequence created by Panaroo for each gene was blasted to the AF2122/97 *M *. *bovis* reference, using a similarity e-value cutoff of 1×10^−10^. A network of strong reference gene – accessory gene BLASTn matches was created using the IRanges R package v2.24.1, and this network was filtered to contain only gene-reference matches where at least 75 % of the length of the Panaroo-identified gene overlapped with a reference gene. The resulting network was used to identify gene fragments that all mapped to the same reference gene, indicating that each fragment was part of the same original reference gene undergoing pseudogenization. Since we expected to identify gene content variation driven by deletion, we investigated whether certain functional categories of genes were more likely to be pseudogenized. We used PANTHER and the PANTHER GO Slim annotation set to analyse whether certain Gene Ontology groups were overrepresented in genes undergoing pseudogenization compared to core genes [23]. Additionally, as a negative control for detecting pseudogenization, we determined whether any of the pseudogenes identified in this analysis were essential in the H37Rv *M. tuberculosis* reference strain [24]. Recently updated AF2122/97 functional annotations were used to map AF2122/97 annotated genes to orthologous coding sequences in *M. tuberculosis* H37Rv [25].

### Core genome recombination detection

A core genome alignment was constructed using parsnp v 1.2 [26], using the *M. tuberculsosis* H37Rv genome as a reference and outgroup. Parsnp uses muscle [27] to create gene alignments and FastTree2 [28] to create a core phylogeny. Recombination was detected using Gubbins [29] with default parameters and a starting tree created by parsnp using a GTR substitution model. For each gene with detected recombination, whole-contig alignments were created using progressiveMauve [30] and reviewed in Geneious Prime v 2021.2.2 to rule out detection by assembly or alignment error. All regions of the genome that Gubbins determined to be recombinant were excised from the core gene alignment to ensure only regions of clonal evolution were included in the population structure analysis. Tree visualisations were created using iTOL v5 [31] and FigTree v1.4.4 (http://tree.bio.ed.ac.uk/software/figtree/). In the process of creating a core genome alignment, parsnp excises regions of the alignment that are missing from at least one genome. Since this resulting alignment is conservative, some potential recombination events may be excised from the alignment before it is fed to Gubbins. Therefore, in addition to the recombination analysis on the core genome alignment, individual gene alignments that had previously been implicated in recombination events in *M. bovis* [7] were also assessed for recombination using fastGEAR [32]. The individual gene alignments were created using the built-in MAFFT aligner included in Panaroo [22, 33].

### SNP validation analysis

Filtered SNPs were used to validate potential recombination hotspots identified by Gubbins and fastGEAR. SNPs were considered valid if they passed the filtering criteria described in section 5.2. SNP quality was also verified by manually inspecting read mapping files using the Integrative Genome Viewer [34]. The functional consequence of SNPs was assessed using snpEff [35].

### Population structure assessed by core and accessory genes

*M. bovis* population structure was compared using core SNPs and accessory gene content. To analyse population structure using the core genome, principal component analysis (PCA) was applied directly to Boolean vector transformed SNP vectors following the methods presented in Konishi *et al*. [36]. In accessory genes population structure was assessed using PCA on the Jaccard distance calculated using binary gene presence absence patterns using the R package abdiv v0.2.0 [37]. A dendrogram was created from accessory gene clusters using Ward’s hierarchical clustering on the Jaccard distance matrix. All post-processing analysis was done in RStudio 2021.09.0 [38] using R v 4.1.2 [39].

### Outbreak analysis

To investigate the utility of gene presence absence data in outbreak investigation, we analyzed the pangenome of 62 *M *. *bovis* genomes from well-described outbreak that occurred in Minnesota from 2005 to 2012 and was documented in Glaser *et al*. [40]. The outbreak pangenome was assessed using Panaroo and the methods described in the Pangenome analysis section above. In addition to gene presence absence patterns, we also created a core genome phylogenetic tree using the SNP matrix from Table S2 in Glaser *et al*. [40] and IQ-TREE-2 v 2.1.1 [41] with a GTR substitution model. We also used ScarTrek [42] to call indels. Indels were removed if they occurred in both the outgroup, sequences from a Texas outbreak (13–3900, 12–5094 and 12–5090), and the Minnesota sequences. Binary indel presence/absence information was used to create an indel tree with a JC2 substitution model in IQ-TREE-2 [41].

### Investigating pangenome openness

To compare the impact of methodological artefacts on *M. bovis* pangenome analysis, we reanalyzed the genomes included in a recent *M. bovis* pangenome study by Reis and Cunha [6]. We downloaded raw or complete sequence files listed in Supplementary Table 1 of Reis and Cunha [6] from NCBI. We processed the non-complete genomes and annotated all genomes using the sequence assembly and annotation methods described in Sequence assembly and annotation above. We assembled the pangenome using Panaroo and the post-hoc filtering methods described in Pangenome analysis above.

All R scripts and parameters used for other bioinformatic programmes are available on https://github.com/kmceres/Mbovis_pangenome.

## Results

### Sequence assembly

A total of 3626 raw fastq files were downloaded from the NCBI Sequence Read Archive. SNPs were called on all download samples and were used to assign clonal complex lineage labels. After lineage labels were determined, 1781 samples were chosen to be assembled to maximize representation of the known *M. bovis* clonal complexes in our final dataset. After assembly and quality filtering, 1463 whole genome sequences remained. All sequences were near complete (>=90 % completeness), and all sequences had low or moderate contamination (<10 % contamination). We found 949 sequences had no contamination. Assembly quality figures are shown in Supplemental Figure 1.

### Core phylogeny

**Fig. 1.**
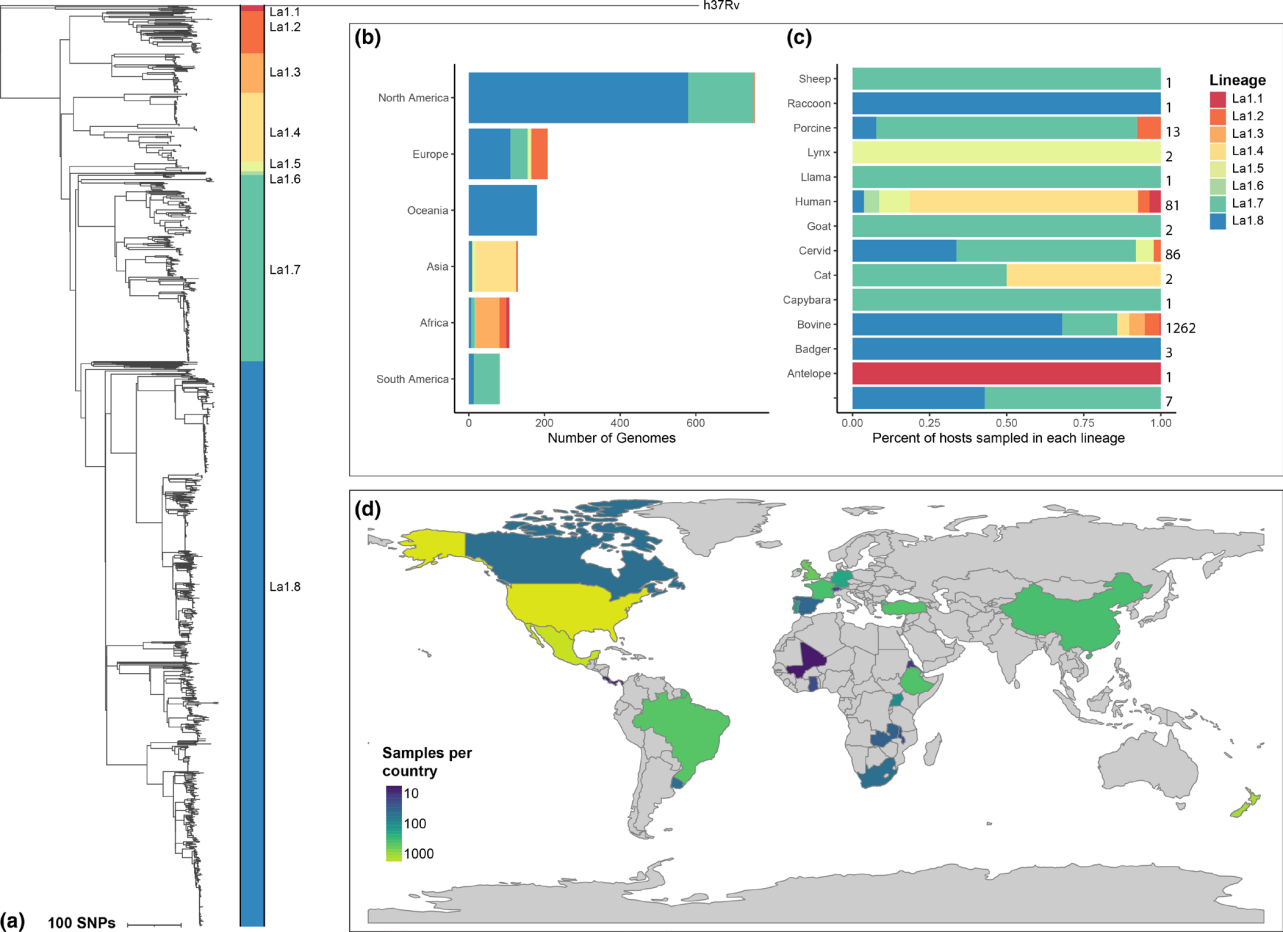
*
M. bovis* core phylogeny and sample geography. A) A recombination-free core phylogenetic tree constructed in Gubbins with a GTR substitution model is shown with clonal complex labels. Sample collection continent distribution is shown in B) and host distribution is shown in C). The number of samples included from each country is shown in D).

The core phylogeny shown in Fig. 1 contained eight clonal complexes labelled using the lineage designations, described in Zwyer *et al.* 2018 [14]. The recombination free core phylogeny was created using RAxML [43] with a GTR substitution model in Gubbins and was rooted at the *M. tuberculosis* reference, H37Rv. Each core phylogenetic group contained samples from multiple geographic regions, reflecting multiple transmission events between geographic regions over the complex history of *M. bovis* evolution [44]. Our sample includes genomes from every continent except Antarctica (Fig. 1c and d), and in a variety of host species (Fig. 1c), however, most samples were from cattle hosts. Lineages La1.7 and La1.8 and samples from the United States, Mexico, and New Zealand were over-represented, reflecting the intensive ongoing bovine tuberculosis molecular surveillance programmes in those regions.

### Population structure analysis

Panaroo identified 3681 core genes present in greater than 95 % of isolates, and 1441 accessory genes including 970 genes present in less than 15 % of isolates. The Panaroo estimate of accessory genome size was smaller than previous studies [4–6, 44–47], which is more consistent with a hypothesis of clonal evolution.

**Fig. 2.**
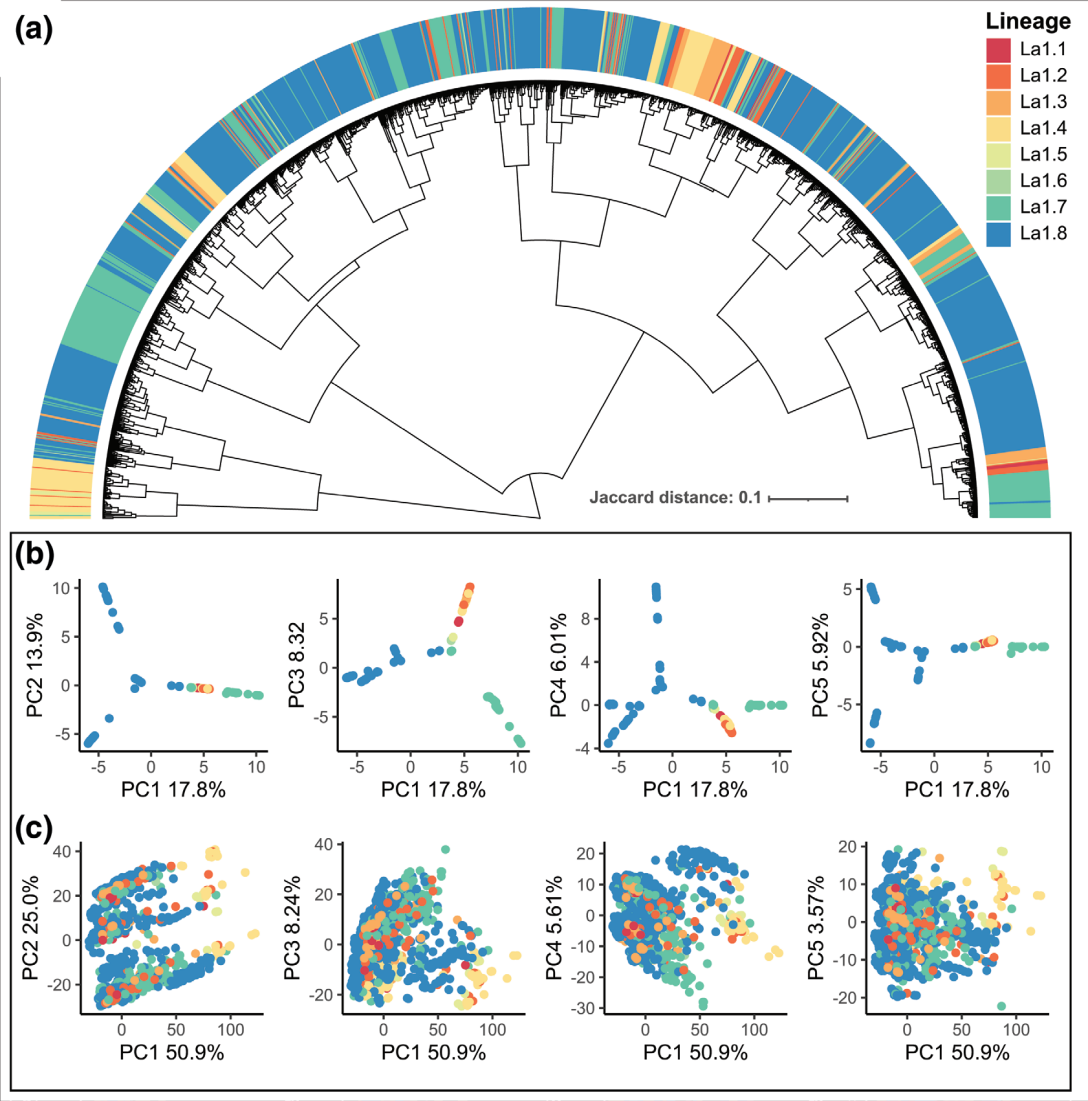
Population structure assessed in SNP variation and gene presence absence patterns. A) Gene content similarity dendrogram created using the Jaccard distance of the pangenome gene presence absence matrix. Core clonal complexes are labelled along the circumference of the tree. B-C) Core SNP and gene content variation principal components analysis coloured by core clonal complexes created with core SNP principal components. SNP variation is shown in B) and gene content variation is shown in C). Gene content variation is not concordant with SNP variation.

The population structure assessed using core SNPs and accessory gene presence absence patterns is shown in Fig. 2. Principal components analysis of the core SNPs and pangenome content are shown in Fig. 2b and c, respectively. A dendrogram was created to further visualize the similarity between gene content variation and core SNP variation. If the pangenome evolved clonally we would expect to see high concordance between core phylogenetic groups and accessory gene content patterns, although incomplete concordance was observed.

To test whether this lack of perfect correlation between genome content variation and core phylogenetic grouping was true evidence of a departure from clonality or if there was classification error generating the discordant signal, we investigated if and where accessory gene consensus sequences mapped to the *M. bovis* reference genome AF2122/97. Only 38 genes did not map to the AF2122/97 reference genome. Two out of the 38 had no blast matches, 29 mapped to *Bos taurus* reference genomes and are most likely host genome contamination, five mapped to non-MTBC microbes, including the *Escherichia* phage PhiX174 and one, *espI_2* mapped to Esx-1 protein EspI on several MTBC genomes including *M. bovis* Mb3601. Similarly, *esxM* did not blast to AF2122/97, but did map to several other MTBC reference genomes. An alignment of *espI_2* between genomes included in this study, AF2122/97, Mb3601 and *M. tuberculosis* H37rv shows that AF2122/97, and many samples included in this study have a 2049 base pair deletion of a duplicated copy of *espI* (Supplemental Figure 2). This one gene deletion is the only example we found of a gene present in our sample but not in AF2122/97, indicating that gene content variation is not generated by the addition of genes from external sources. We cannot rule out that the phage PhiX174 infected some *M. bovis* genomes, however all contigs that contained genes that mapped to PhiX174 did not contain any *M. bovis* genes, suggesting the sequence fragments containing PhiX174 genes were not part of *M. bovis* genomes. Since *M. bovis* does not contain plasmids, it is more likely that these partial phage genes were remnants of the standard Illumina phiX internal sequencing control that did not get fully removed as part of their automated demultiplexing process. Because we did not find genes contained in *M. bovis* genomes that did not map to a *M. bovis* reference genome, it is unlikely that *M. bovis* acquires new genes from other prokaryotes via HGT.

### Redundant labeling of accessory genes

**Fig. 3.**
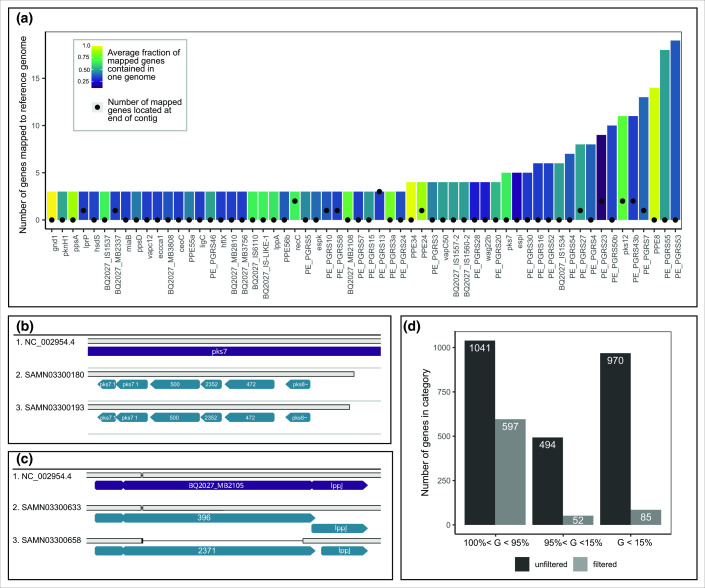
Accessory gene labels are created through redundant gene annotation. A) The distribution of all redundantly labelled genes, coloured by the fraction of the time all genes in a redundant gene annotation group were found in the same genome. Within each reference genome, the number of mapped genes that were found within 1000 bp of the end of a contig are shown (black circles). B) and C) Alignments of two example genes, *pks7* and BQ2027_MB2105 (purple) with redundantly annotated accessory genes (blue). D) After removing redundant gene annotations, the accessory genome size (genes present in less than 95 % of genomes) decreased from 1464 to 137 genes.

Further inspection of alignments of contigs with and without accessory genes showed that most accessory genes were redundant annotations of the same gene undergoing pseudogenization. A subset of redundant gene groups with at least six genes mapped to the same reference genome are shown in Supplemental Figure 3. The gene presence absence pattern of each redundant gene group is plotted along the circumference of the core phylogeny and is coloured by the reference gene to which the redundant gene group maps. There are two distinct patterns of gene content variation among redundant accessory genes. Either the redundant genes are mostly found on the same genome, or the genes within a group are more diffusely present or absent in genomes. This gene content variation pattern was further explored in Fig. 3a, which shows the distribution of gene groups with at least two redundant genes mapped to a single reference genome, coloured by the fraction of the time that all of the genes in a given group were found on the same genome. A total of 29 out of the 60 reference genes that had three or more mapped redundant gene groups were PE and PPE family genes. PE/PPE genes are highly repetitive and have an increased number of indel mutations compared to genes with higher sequence complexity [42]; therefore, it is likely some of the redundant annotation is due to frameshift mutations caused by indels. The low sequence complexity of PE/PPE genes also makes them harder to sequence accurately, so a fraction of the redundant annotation may also be attributed to sequencing or assembly error. Because PE and PPE gene families are difficult to sequence accurately, they are often excluded from molecular epidemiological analyses [48].

Example gene alignments demonstrating the two causes of redundant annotation are shown in Fig. 3b and c. The accessory genes in B were caused by annotation error, likely due to low coverage at the 3′ end of *pks7*. In C, the difference in accessory gene labelling was caused by a deletion in BQ2027_MB2105 in a subset of genomes including SAMN03300658. This deletion led to the annotation of an additional gene, labelled 369. An additional source of annotation error resulted from palindromic contigs, which likely resulted from assembly error. Although we removed palindromic contigs through visual inspection described in the Sequence assembly and annotation section, additional palindromic contigs remained in the analysis. The result of palindrominc contigs was identical sets of genes sequence annotated with different gene names. On non-palindromic contig genes from the identical sets received either the original gene annotation or the reverse compliment annotation. This resulted in an inflation of accessory genome count because core genes were designated as accessory due to inconsistent annotation. When we removed redundant gene labels generated by mislabeling at the ends of contigs, and palindromic contig labels, the size of the accessory genome decreased from 494 accessory genes present in between 15 and 95 % of genomes and 970 rare genes present in less than 15 % of genomes to 52 accessory and 85 rare genes (Fig. 3d). The remaining accessory genes were either paralogs (33 genes) or pseudogenes (104 genes).

Although these redundantly annotated genes do not represent true accessory gene content variation, they do represent true differences in genotypes. This set of redundant genes contains genes with frameshift mutations leading to premature stop codons and loss of gene function, including mutations in single base repeats creating homopolymeric tracts, that may be reversible [42, 49]. We found insertions and deletions in homopolymeric tracts in multiple genes including *frdBC* and a C537, 7C→8C insertion in *glpK*, which was shown to cause reversible antibiotic tolerance in *M. tuberculosis* [49]. Interestingly, the frameshift-causing homopolymeric tract mutations in *glpK* are roughly uniformly distributed across the phylogeny, indicating that these mutations occur in the absence of a clear selective force (Supplemental Figure 4A). An example alignment of the variable homopolymeric region in sequences from an outbreak that occurred in New Mexico, which had variable presence or absence indicating that variation in homopolymer tract length occurred within relatively short timespans (Supplemental Figure 4B).

### Attributes of accessory genes

**Fig. 4.**
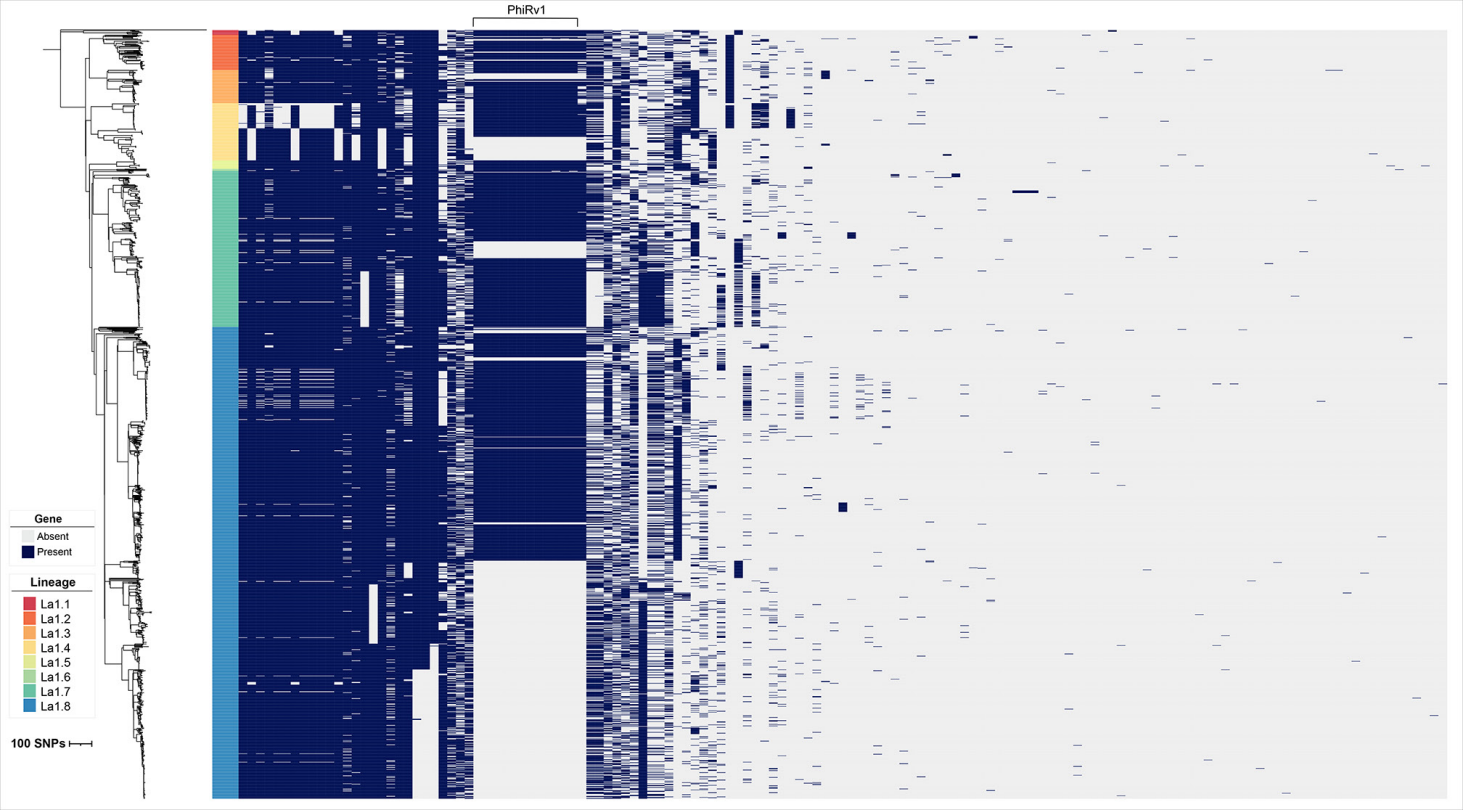
Filtered gene presence absence patterns in the *M. bovis* pangenome. The core genome phylogeny from Fig. 1 is shown, labelled with clonal complexes. The phage PhiRv1 genes are also labelled. Branch lengths are in SNP units.

The gene presence absence patterns of the filtered set of accessory genes are shown in Fig. 4. Three patterns of gene content variation were observed. First, it is evident that eight accessory genes have perfectly correlated presence and absence patterns, shown in Fig. 4. These genes are a group of eight pseudogenes that are remnants of a phage, PhiRv1, also identified in the *M. bovis* accessory genome by Zimpel *et al*. [44], which was thought to be acquired when the ancestor of the MTBC transitioned to an intracellular lifestyle. This phage is absent in the *M. bovis* BCG vaccine strain, and it is hypothesized that the loss of this phage is associated with the reduced virulence of the vaccine strain [50]. PhiRv1 genes are present in most lineages but are notably absent from a monophyletic group within La1.8 consisting of 49 % of the La1.8 samples included in this study. Absence of PhiRv1 genes from this subgroup could be used as a phylogenetic marker. We ran an additional PCA analysis on the Jaccard distance of the filtered presence/absence matrix and found that there were two main clusters of accessory genes. Although accessory genes do not appear to cluster by core genome lineage (Supplemental Figure 5A), the two distinct PCA clusters are separated by the presence and absence of the PhiRv1 genes (Supplemental Figure 5B). The second group consists of genes identified to be paralogs by Panaroo. This group consists mainly of genes involved in transposition (transposases, endonucleases), but also contains PE/PPE family genes and four genes that form a unique cluster in *M. bovis* La1.7. These genes originated from a single, duplication event of four genes (*bpa*, *ragH*, *glfR*, and a hypothetical protein) within *rfbB* (Supplemental Figure 6). The last pattern of accessory gene content is more diffuse presence or absence, and this pattern is driven by accessory genes that are undergoing pseudeogenization. We were not able to identify any functional category that was more likely to be undergoing pseudogenization in the PANTHER overrepresentation test, suggesting that the process of gene pseudogenization may be somewhat random; however, essential genes seem to be protected from pseudogenization. Only one detected pseudogene mapped to an essential *M. tuberculosis* H37Rv gene, *glmS*. Although 14 genomes contained this pseudogene, each genome had a different insertion sequence all located in a repetitive region of *glmS*; This region of *glmS* may either be hypervariable or difficult to sequence accurately.

### Gene content variation in outbreaks

**Fig. 5.**
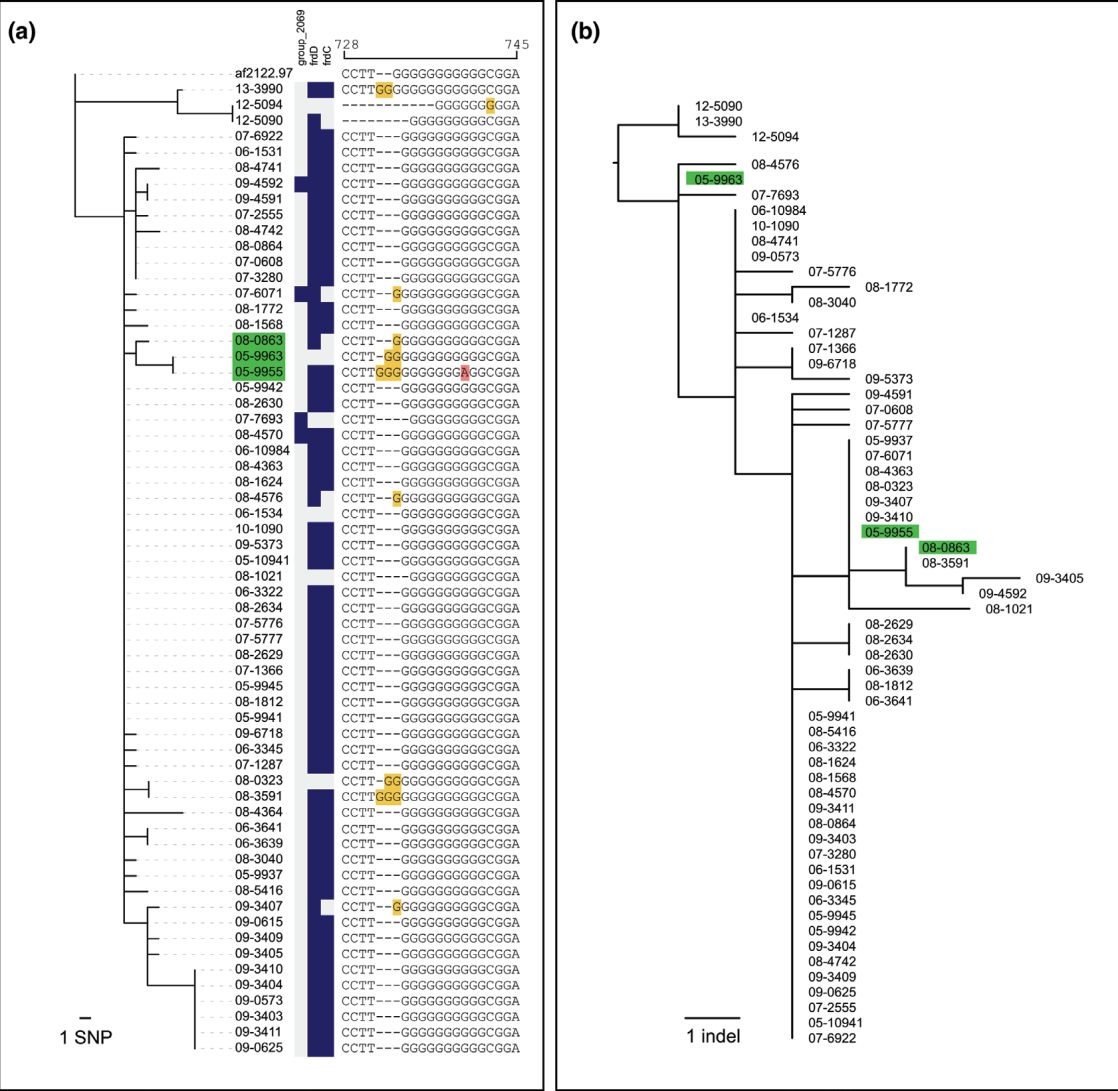
Gene content variation in a Minnesota outbreak. Figure A shows a core genome phylogeny created with IQ-TREE2 using a GTR substitution model, and a gene presence absence matrix. Variation in gene content is observed in three genes, not including PE/PPE genes, however all gene content variation was driven by indels. An alignment of a variable homopolymeric tract region of *M. bovis* AF2122/97 *fdrBC* is shown next to the gene presence absence matrix. This homopolymeric tract variation resulted in differential gene presence absence annotation of *frdD* and *frdB* genes among outbreak samples. Indel variation is shown in a tree created with binary indel presence/absence data in IQ-TREE2 with a JC2 substitution model in B. Three samples are highlighted to show disparate topologies of the SNP and indel trees. Three samples that had conflicting phylogenetic positions are highlighted in green.

Variable gene content over an outbreak in Minnesota is shown in Fig. 5a next to a core genome phylogenetic tree. The tree is rooted at the common ancestor to a closely related outgroup of sequences from a Texas outbreak (13–3900, 12–5094 and 12–5090) as in Glaser *et al*. [40]. Only three genes were found to be variable over this outbreak, group_2069, *frdC* and *frdD*. Upon further inspection of contig alignments, group_2069 was found to be annotated in genomes that lacked a 60 bp deletion between *thrB* and *thrC* compared to the reference genome AF2122/97. Due to the sparse distribution of group_2069 among outbreak samples, a more parsimonious explanation may be that the genomes with group_2069 have a 60 bp insertion relative to the other outbreak genomes, which created a new open reading frame. The inserted sequence is identical to an adjacent 57 base pairs in the *thrB/C* intergenic region, suggesting this sequence was duplicated in genomes with group_2069 annotated. The other two genes, *frdC* and *frdD* were components of the AF2122/97 genes *frdBC* and *frdD* which were annotated differently depending on the insertion or deletion of base pairs in the homopolomyeric tract of *frdBC*. An alignment of the sequence differences in the *frdBC* homopolymeric tract are shown next to the gene presence absence matrix in Fig. 5a. All gene content variation that was identified in the pangenome of the outbreak sequences was driven by indels; however, not all indels that occurred during the outbreak were identified in the pangenome analysis since only indels that changed an open reading frame were noted as variable gene content. Fig. 5b shows the relationships of indel presence and absence of outbreak sequences detected by ScarTrek [42]. As expected, there is more variation in indels than was captured in the gene presence absence analysis.

**Fig. 6.**
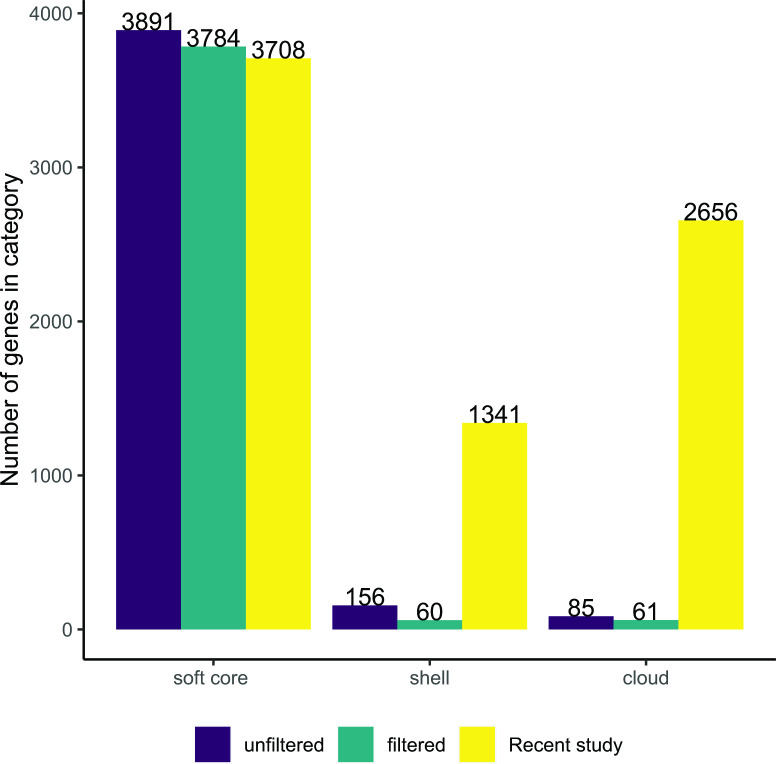
Methodological differences result in drastically different pangenome sizes. Panaroo produced a smaller accessory genome than that described in a recent *M. bovis* pangenome study. Soft core genes are present in greater than 95 % of samples. Shell genes are present in less than 95 % of samples but more than two samples. Cloud genes are present in one or two samples. After filtering redundantly labelled genes, the pangenome size was further reduced. The *M. bovis* pangenome is not open and shows presence and absence patterns consistent with clonal evolution.

Glaser *et al*. included two different phylogenetic analyses [40]. The first (Glaser *et al*. Fig. 6) was a maximum likelihood phylogeny resulting in the same tree as shown in Fig. 5a, where two cattle samples from the same herd (05–9955 and 05–9963) were placed in a clade with a deer sample (08–0863) because these three samples shared one SNP absent in other samples. The second tree included temporal information and produced a Bayesian time calibrated tree which placed two samples from the same cattle herd (05–9955 and 05–9963) at the base of the tree (Glaser *et al*. Fig. 5), and deer sample in a distant clade. The second tree would suggest that the cattle and deer samples were not closely related and perhaps were not linked in a transmission event. Interestingly, when comparing the SNP tree to the indel tree in Fig. 5, there is another difference in these three samples shown in these highlighted samples in Fig. 5. The positions of these three samples on the indel tree in Fig. 5b adds an additional dimension to the two phylogenetic analyses presented in Glaser *et al*., showing cow sample 05–9963 and deer sample 08–0863 share an additional indel not present in 05–9955. The combined analysis of SNPs and indels suggests that these samples may be more closely related than was suggested in the timed tree. In conclusion, although true gene content variability may occasionally occur during outbreaks in the form of duplication or larger gene deletion events, indels are likely more common, and may be useful in outbreak investigation when few SNP differences exist among samples.

### Recombination

To determine if there is evidence of ongoing HGT in the core genome, we used Gubbins to infer recombination history in the core gene alignment and fastGEAR to detect recombination in a selected set of individual gene alignments. Gubbins inferred 16 recombination events; however, only five occurred on internal branches. Recombination events inferred on terminal branches can be unreliable because they only involve one genome. In addition to recombination analysis done on the core genome alignment, fastGEAR was used to investigate recombination on individual gene alignments with recombination events implicated in Reis and Cunha [7], and to confirm the recombination events detected by Gubbins. The fastGEAR analysis confirmed recombination events identified in Reis and Cunha [7] in *narX* and *pks12*. There is a high density of SNPs in a 100 bp locus in *narX* in18 monophyletic genomes. An alignment of the genomes with the *narX* SNP hotspot is shown in Supplemental Figure 7A. Four of the SNPs are nonsynonymous, resulting in three amino acid changes. An additional potential recombination event involving a 16 SNP hotspot was identified in *pks12*. The *pks12* SNP cluster is also found in a monophyletic group, shown in Supplemental Figure 8A and B. SNPs occurred at the 3′ end of *pks12* directly adjacent to regions of consistent poor mapping quality. In fact, the region of *pks12* that contained the SNP hotspot was always located at the end of a contig in our draft genomes (Supplemental Figure 8C). Since the SNP hotspot occurred at the end of contigs, near gaps, we could not confirm with high certainty that the SNPs were not called erroneously. Gubbins and fastGEAR also identified three additional recombination events in PPE 21, ppsB, and esxN. These potential recombination events consisted of sets of between five and seven SNPs, each shared by a monophyletic group.

### Pangenome openness

To determine the impact of methodological differences on *M. bovis* pangenome representation, we re-constructed the pangenome using the 70 genomes used in Reis and Cunha [6]. The counts of accessory genes in the original analysis done by Reis and Cunha along with the Panaroo results before and after filtering are shown in Fig. 6. Even before post-hoc filtering, the Panaroo estimate of accessory genome size was much smaller compared to the estimate obtained previously. Reis and Cunha used the get-homologs pipeline to analyse the pangenome [51], which unlike Panaroo does not employ a draft genome annotation correction method to reduce false positive accessory gene annotations. Using our methodology, we can reject the concept that the *M. bovis* pangenome is open, since we identified only 121 accessory genes in the 70 genome dataset, compared to the Reis and Cunha estimate of 3997 gene clusters. The gene presence/absence matrix of the pangenome created with the Reis and Cunha dataset is available in Supplemental File 3.

## Discussion

Since a single genome contains only a portion of the genetic diversity of a species, comparing a new set of sequences to just one reference genome alone provides an incomplete description of the true genetic diversity in the set. There are currently hundreds of thousands of bacterial genomes publicly available on GenBank and other sequence repositories, potentially enabling the characterization of the full range of gene content diversity in multiple species; however, technical advances in annotating draft genomes have not caught up with the enormous number of incomplete genomes being uploaded to public databases [52]. One reason draft genomes are difficult to annotate is simply that genomes are incompletely sequenced and contain gaps between contigs. If the average prokaryotic gene is on the order of 1 kb long, any contig with length on the same order of magnitude will likely contain fragmented genes. The ends of longer contigs also likely contain fragmented genes. Unfortunately, the only solution to this problem is to improve the sequence quality by closing the gaps between contigs, which perhaps will be done with future genomes sequenced using long read technology but may be too time intensive and costly for the thousands of existing draft genomes to be re-sequenced [52].

Methods for characterizing the pangenome typically involve identifying and classifying clusters of orthologous genes into core and accessory genes, without specifically accounting for erroneous annotations. Panaroo attempts to correct annotation errors by creating a graphical representation of the pangenome and employing several quality control steps to remove poorly supported nodes [22]. With these additional quality control steps, Panaroo was able to more accurately characterize the *M. tuberculosis* pangenome compared to several other pangenome pipelines [22], however correctly characterizing gene fragments remains an issue.

Another perhaps unavoidable issue in pangenome studies is sampling bias. Since bacterial population sizes are so vast, even a relatively large sample may not truly represent the diversity in gene content present in a species [53]. Additionally, although we downloaded thousands of *M. bovis* genomes available from the NCBI Sequence Read Archive, we only retained the highest quality assemblies for analysis to minimize errors described above caused by higher contig number assemblies. The resulting sample still represents a convenience sample of *M. bovis* outbreaks in countries with well-funded surveillance programmes, and may not fully represent *M. bovis* variants present in cattle globally. However, our sample includes genomes from all known major *M. bovis* clonal complexes and may be representative of the true diversity of the geographic regions included in this study because of intensive sampling programmes in those regions.

Despite the limitations in data quality created by short read sequencing, we were able to determine that *M. bovis* likely evolves clonally. After correcting for redundant gene annotations, we found a small pangenome consistent with a general pattern of ongoing gene deletion resulting in an increasingly compact genome. This gene deletion appears to be driven by random indel-causing pseudogenization. We also show that the *M. bovis* pangenome is likely closed, contradicting recent work by Reis and Cunha suggesting the *M. bovis* pangenome is open [6].

Additionally, we determined that accessory gene content is generally stable during outbreaks, although some variability in gene content is observed even between closely related individuals. Since *M. bovis* and other MTBC species have a slow mutation rate (less than one substitution per genome per year) [54], genomes sampled in outbreak investigation often have very similar or identical SNP patterns. This low SNP diversity makes inferring who infected whom to be difficult or impossible using SNP data. Gene content variation could occasionally be useful in outbreak analysis to discriminate between samples with very similar SNP patterns, although variability in accessory genomes among closely related outbreak sequences is low. One important caveat in using gene content variation in outbreak analysis is that if accessory genes are not carefully examined, inferences on transmission patterns based on gene content variation are likely to lead to incorrect conclusions because of the inherent problem of gene detection in incomplete genomes. Indel presence/absence may be a more reliable and more common source of sequence variation in outbreak investigations. Using both indel and SNP data to infer transmission patterns may result in higher resolution transmission networks.

We also identified frameshift mutations in *glpK* and in similar homopolmeric tracts in other genes that may be related to antimicrobial resistance in *M. tuberculosis* [55]. Recently, Safi *et al*. demonstrated that nucleotide insertions in homopolymeric C tract in *glpK* lead to the gene transient gene inactivation, which led to reduced growth under nutrient deficient conditions or antibiotic treatment [49]. After relieving the stress caused by nutrient deficiency or antibiotic treatment, the inserted base pair was removed and colonies were able to restart growth. Therefore, Safi *et al*. conclude that this phase variation represents an additional mechanism for the evolution of antibiotic tolerance and could bias evolutionary trajectories toward antimicrobial resistance. Interestingly, this same C537, 7C→8C insertion was identified in our dataset. Since cattle are not treated with antimicrobials for bovine tuberculosis, *glpK* phase variation in *M. bovis* may not arise as a compensatory mechanism for antibiotic tolerance, but instead may result from drift. In this case, under conditions of antibiotic use, bacteria with the frameshift mutation would have a selective advantage and increased survival. The *glpK* gene encodes glycerol kinase, which is necessary for glycerol catabolism; however, most *M. bovis* strains have an inactivating mutation in *pyk*, the gene that encodes pyruvate kinase, which prevents *M. bovis* from being able to use glycerol as a sole carbon source [56]. Therefore, *glpK* may be dispensable in *M. bovis* and inactivating mutations may arise due to relaxed negative selection or even positive selection to limit the energetic cost of protein production for an enzyme that is not required [57]. More work is necessary to understand the role of phase variation in MTBC evolution, but evidence of *glpK* phase variation in *M. bovis* in cattle not treated with antibiotics for bovine tuberculosis suggests phase variation, especially in non-essential genes, could be a basal evolutionary process that occurs in the absence of antibiotic selection pressure. Indel mutations, generally, may be a random mutation process that selection can act on in addition to SNPs. Recently indels were implicated in antimicrobial resistance in *M. tuberculosis* [58], and it is possible that those resistance-associated indels first arose due to drift, and then were selected due to antimicrobial use.

Lastly, we show that recombination is not an important driver of evolution in the *M. bovis* core genome. Recently Reis and Cunha presented compelling evidence that a low level of recombination does occur in *M. bovis* [7], and we were able to replicate their findings in one gene, *narX*, and detected potential recombination events in an additional three genes; however, the events in PPE 21*, esxN*, and *ppsB* only contained five to seven SNPs. Although it is possible that these mutations were caused by homologous recombination, other evolutionary forces such as recent positive selection could create a mutation hotspot [59]. Furthermore, all genomes with potential recombination events are part of monophyletic lineages, so it is likely that all mutations were caused by a single event. However, the mutation pattern in *narX* is unusual, and from this analysis alone we cannot rule out that it was caused by homologous recombination.

Together, the small accessory genome and lack of significant evidence of recombination, provides strong evidence against the hypothesis that there is ongoing HGT in *M. bovis *, and instead, *M. bovis* indeed likely evolves strictly clonally. This finding has important implications both in understanding the basic mechanisms of MTBC evolution and in the practice of using genomic data in outbreak investigation. Strict clonality restricts the ability of *M. bovis *, and likely all MTBC species, to rapidly acquire genes that would allow it to adapt to new environments, including genes that would promote antibiotic tolerance and resistance. Although antimicrobial resistance is a growing global concern in MTBC and *M. tuberculosis* in particular, strict clonality implies that all antimicrobial resistance must evolve through *de novo* mutations. In outbreak analysis, SNP based bifurcating phylogenetic trees can be used reliably, without the need for complex graph structures to understand transmission.

## Conclusions

*M. bovis* is likely a strictly clonally evolving pathogen, and most variation in gene content is driven by gene deletion. *M. bovis* pangenome evolution is trending toward an increasingly compact genome, leaving only genes that are necessary for life inside the host. We also show that most recombination events and accessory genes identified by bioinformatic programmes were false positives, although Panaroo’s graph connection mechanism appears to outperform other pangenome characterization algorithms in reducing the number of false positive genes. Because of the inherent problem of missing data created by using incomplete sequences, and the inability for bioinformatic programmes to detect and rectify all errors in input data, both accessory gene and recombination events detected using incomplete MTBC genomes must be scrutinized to avoid incorrect inference based on pangenome data. We also found potentially reversible homopolymeric tract mutations in multiple genes, providing evidence that phase variation may be a general mechanism of gene regulation in MTBC. Lastly, we show that a small amount of gene content variation evolves over short time periods among closely related sequences, and this gene content variation is driven by indels. Indels may be a useful source of sequence variation in outbreak analysis when discriminating between genomes with similar SNP patterns.

## Supplementary Data

Supplementary material 1
