# Supplementary material for: A critical evaluation of Mycobacterium bovis pangenomics, with reference to its utility in outbreak investigation
Source: Microb Genom. 2022 Jun 28;8(6):mgen000839. doi: 10.1099/mgen.0.000839 (PMC9455707; doi:10.1099/mgen.0.000839)
Supplement: Supplementary material 1 [file mgen-8-839-s001.pdf]

Supplementary Materials

A critical evaluation of *Mycobacterium bovis* pangenomics, with reference to its utility in outbreak investigation

Kristina M. Ceres[1,2*], Michael J. Stanhope[1,2] & Yrjö T. Gröhn[1,2]

1. Department of Population Medicine and Diagnostic Sciences, College of Veterinary Medicine, Cornell University
2. Population and Ecosystem Health

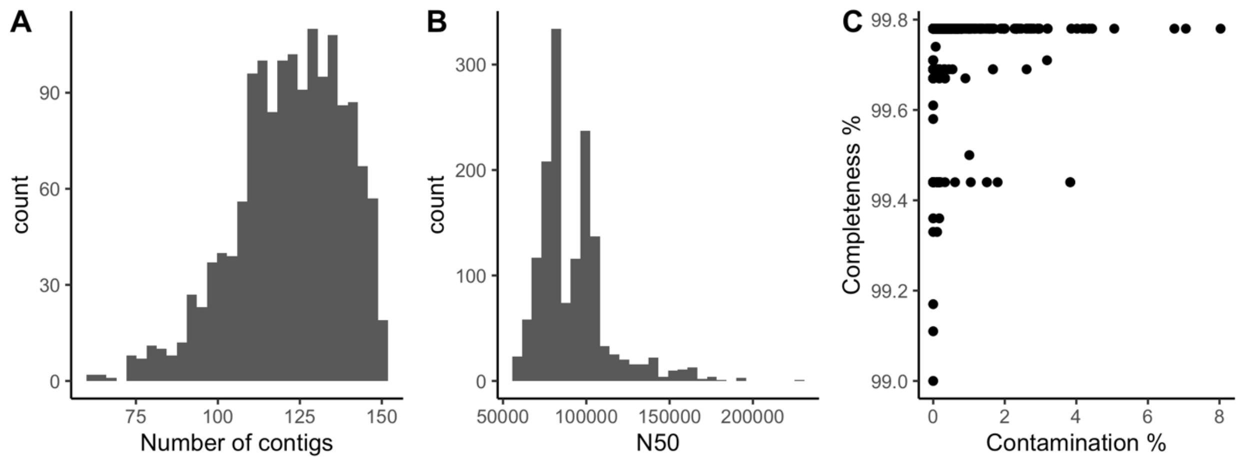

Supplemental Figure 1 **A.** Distribution of contig counts and **B.** N50 among genomes used in this study. **C.** Percent completeness and percent contamination of each genome as detected by checkM.

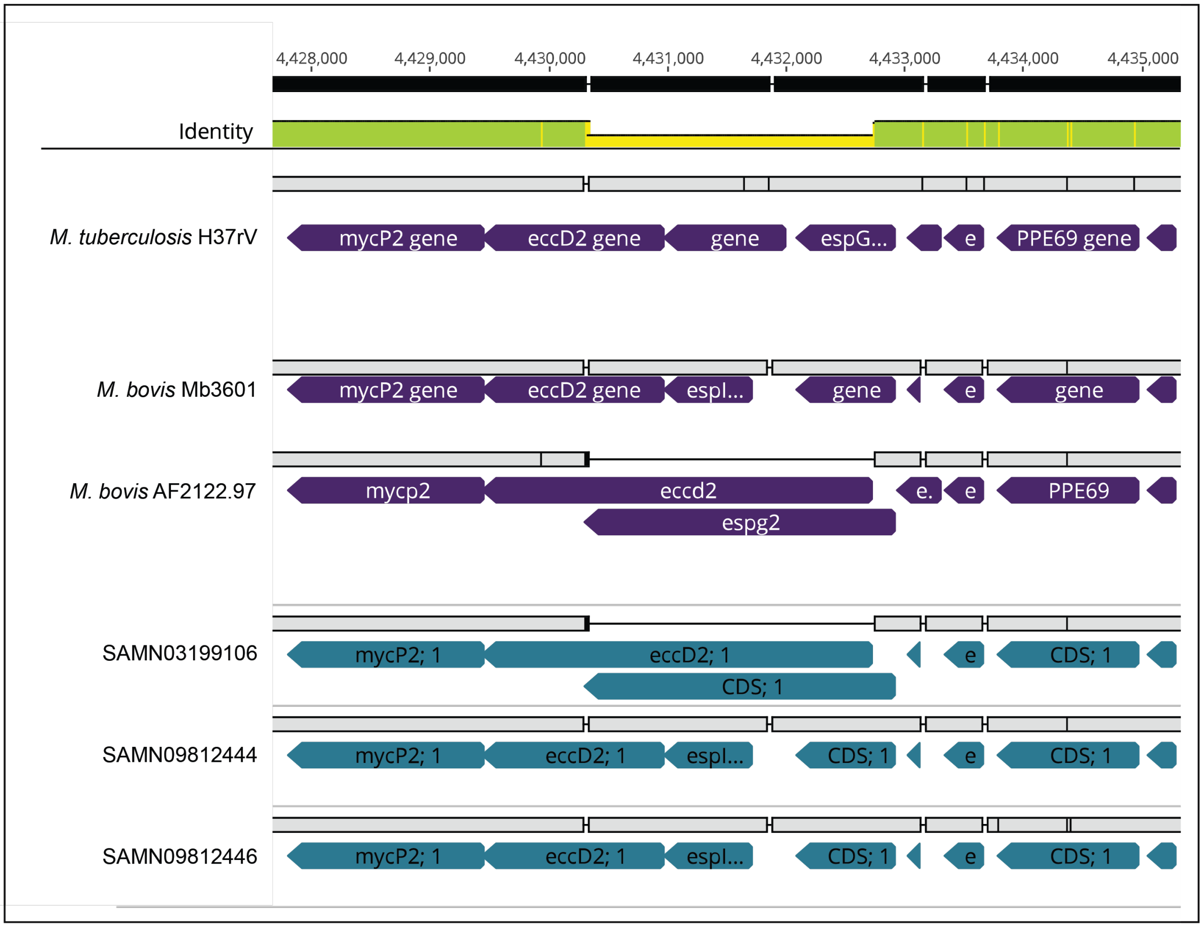

Supplemental Figure 2. EsxI paralog is absent from reference genome AF2122.97 but present in M. tuberculosis H37rV, *M. bovis* Mb3601 and 173 genomes in this study. Of the 173 genomes with the EsxI paralog present, 75 were found in La1.2, 63 in La1.3 and 43 in La1.4.

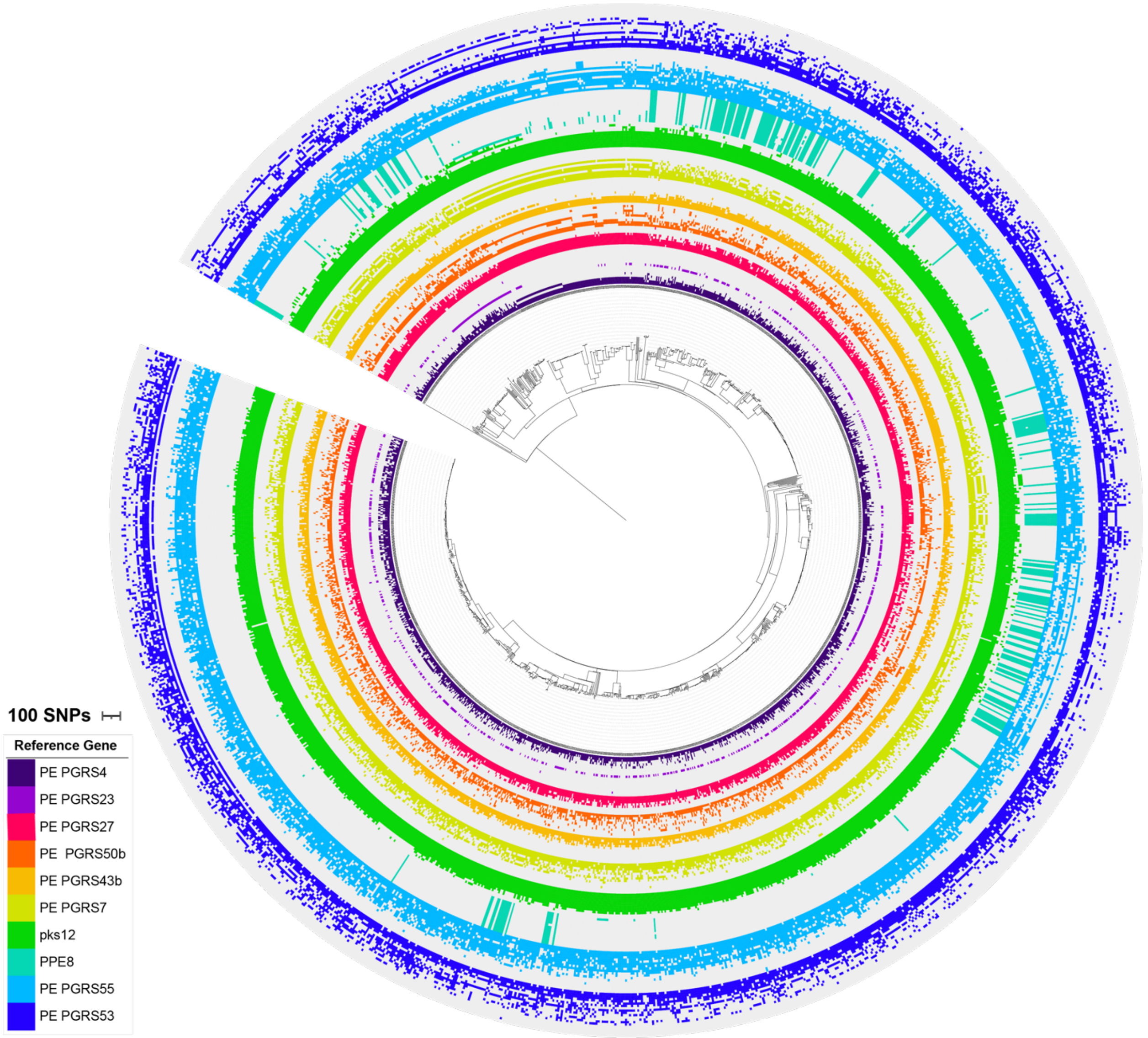

Supplemental Figure 3. The distribution of redundantly annotated accessory genes. The circular core phylogeny shown in Figure 1 is labeled with a gene presence absence matrix of redundant gene groups consisting of 6 or more genes labeled by mapped reference gene on the circumference of the phylogeny. Accessory genes are either found on the same genome such as genes mapped to PPE8, or are more diffusely present or absent.

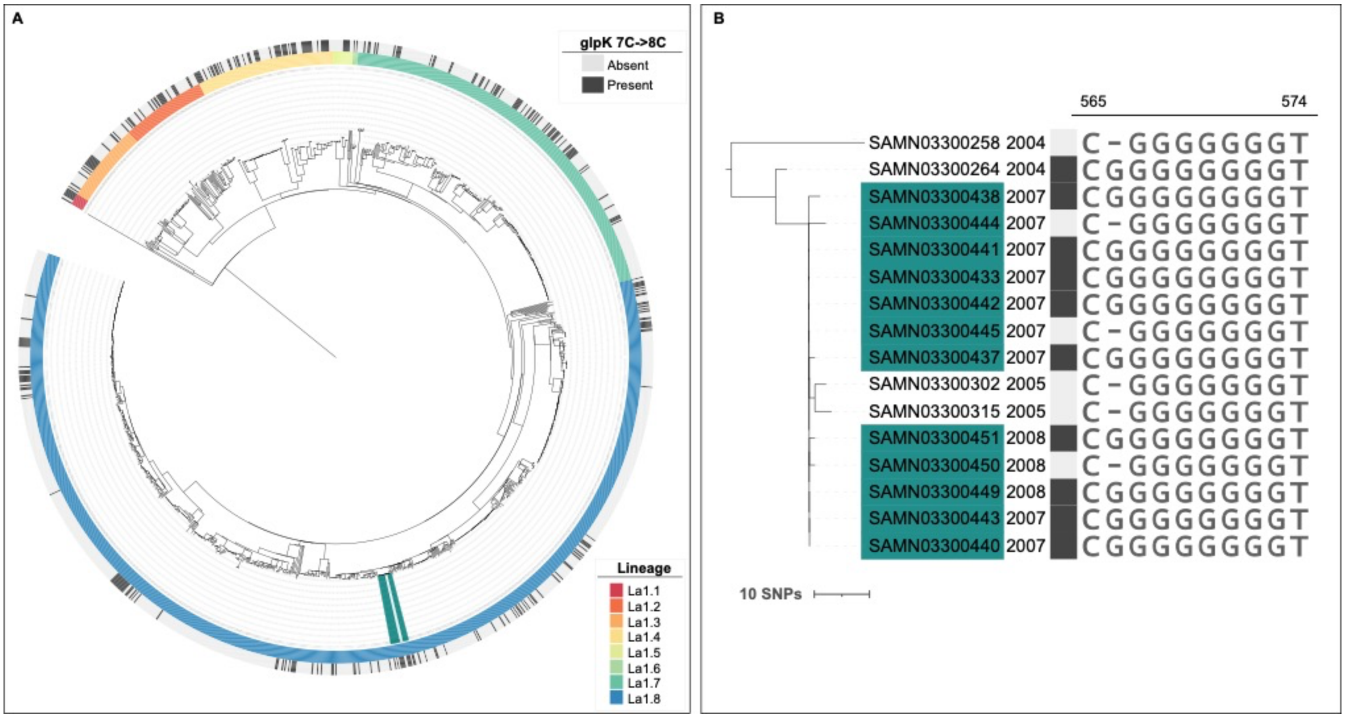

Supplemental Figure 4. The distribution of *glpK* homopolymeric tract insertions. A. The distribution of 7C->8C insertions in *glpK* distributed on a circular representation of the core phylogeny shown in Figure 1. B. Variable homopolymeric tract lengths were found in closely related sequences from an outbreak in New Mexico labeled in teal, which occurred between 2007 and 2008. The homoplomyeric tract mutations appear to be transient.

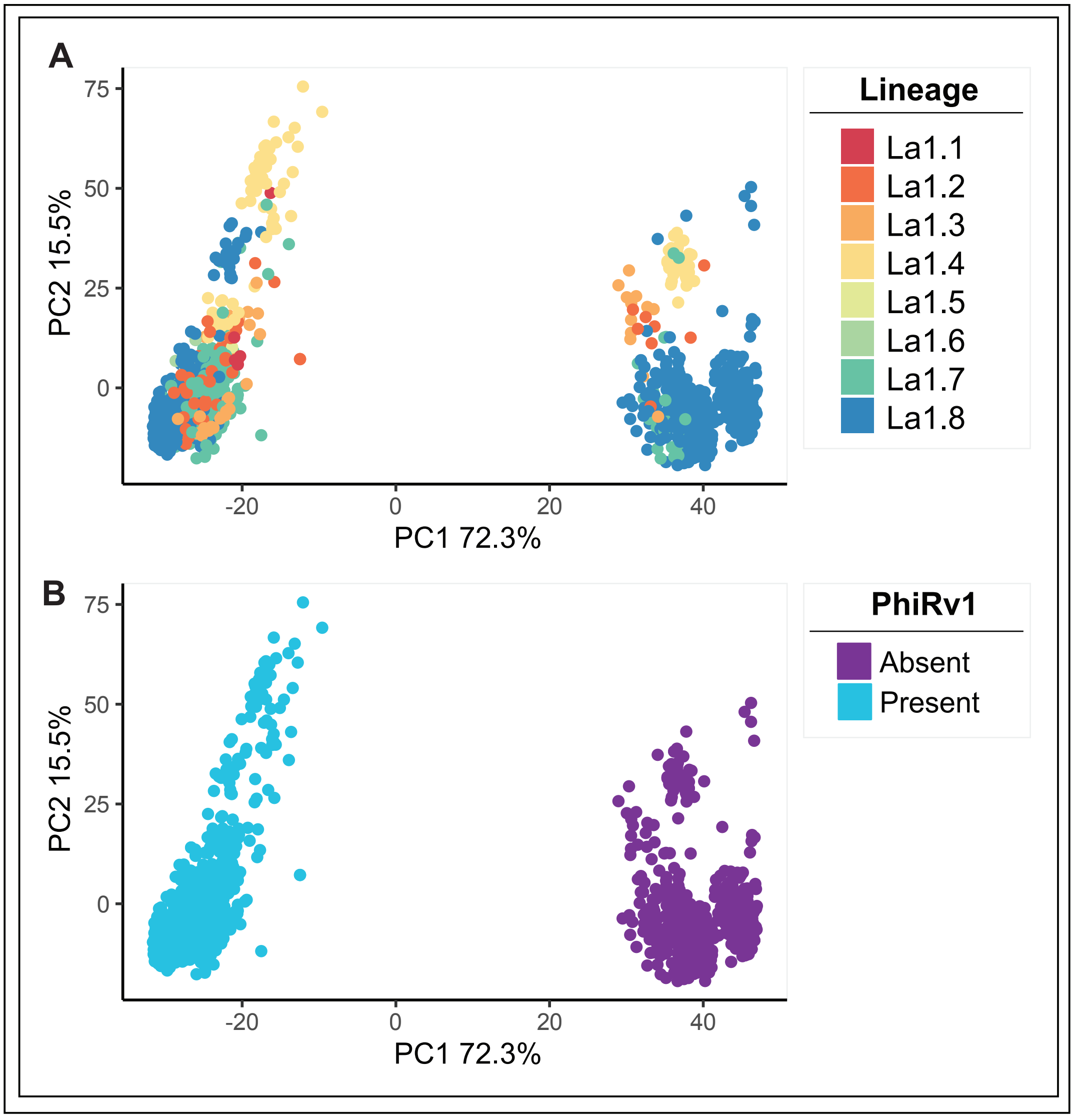

Supplemental Figure 5. PCA of the Jaccard distance of the filtered gene presence absence matrix. In A. points are colored by Lineage, and in B. points are colored by the presence or absence of the phage PhiRv1 genes. The percent variation described by each principal component is shown on the x and y axes.

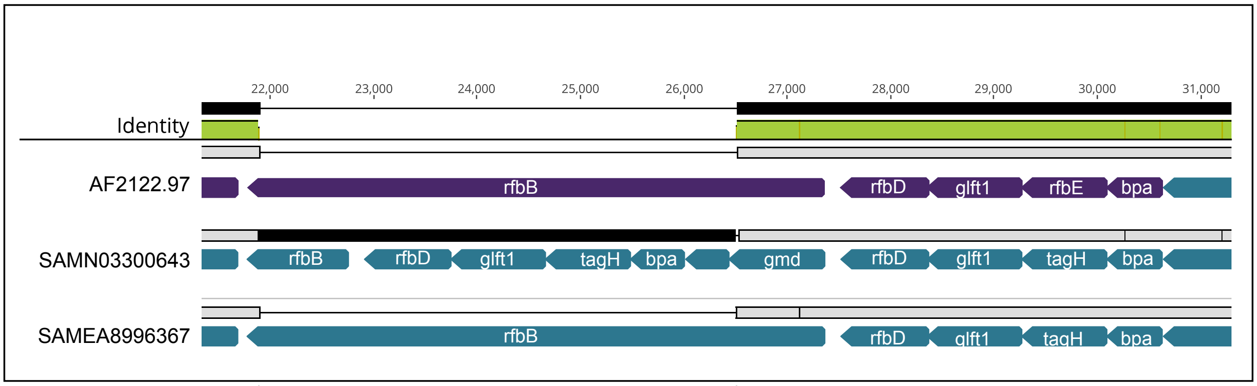

Supplemental Figure 6. Gene duplication. Genes were duplicated in 84 Lineage La1.7 genomes. A representative genome, SAMN03300643 has the 4 gene duplication within rfbB compared to the reference genome (labeled in dark purple), and genome SAMEA8996367.

Reference gene	Previously detected	Gubbins detected	FastGEAR detected	SNPs validated	Event
narX	Yes	No	Yes	Yes	14 SNPs (18 monophyletic genomes)
pks12	Yes	Yes	Yes	Yes	17 SNPs (151 monophyletic genomes)
pks12	Yes	Yes	Yes	Yes	17 SNPs (4 monophyletic genomes
espa	T	No	Yes	No	Ends of contigs
tatA	Yes	No	No	NA	NA
PE PGRS22	Yes	No	No	NA	NA
rrs	Yes	No	No	NA	NA
PPE21	No	Yes	Yes	Yes	5 SNPs (7 monophyletic genomes)
esxN	No	Yes	Yes	Yes	5 SNPs (56 monophyletic genomes)
ppsB	No	Yes	No	Yes	7 SNPs (2 monophyletic genomes)

Supplemental Table 1. Recombination events identified by Gubbins or fastGEAR. SNPs were validated if they were detected both by a recombination detection program and were identified SNPs by the vSNP pipeline after quality control filtering. Previously detected refers to the recombination events detected in Reis & Cunha, Scientific Reports 2021.

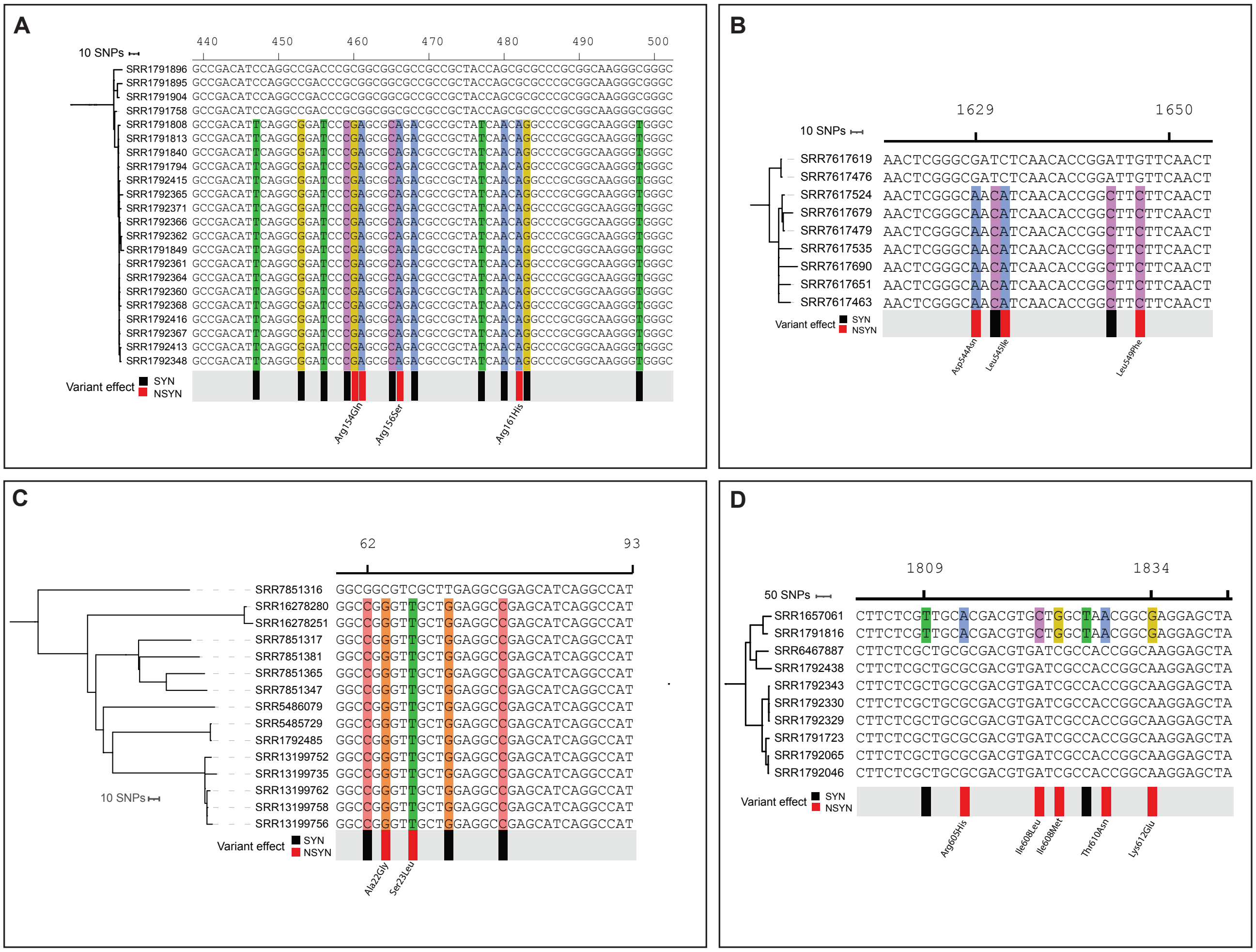

Supplemental Figure 7. Potential recombination events confirmed by fastGEAR in A. *narX*, B. PPE 21, C. *esxN*, and D. *ppsB*. A. The recombinant segment in *narX* contains a hotspot of 14 SNPs shared by 18 sequences from a single outbreak. Four SNPs are nonsynonymous and result in three amino acid changes. B. In PPE 21, three mutations were nonsynonymous. C. Two out of the five mutations were nonsynonymous, and in D. five out of the seven mutations were nonsynonymous.

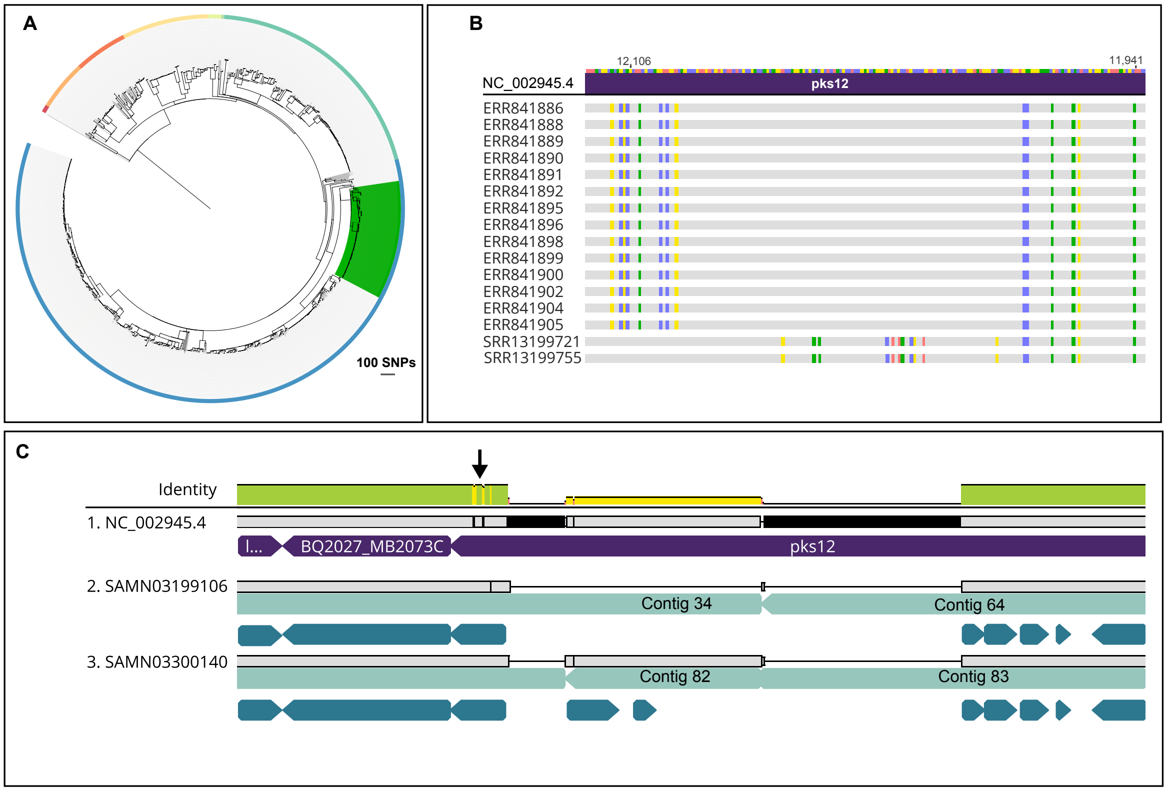

Supplemental Figure 8. SNP hotspot in *pks12* is not verified because of low mapping quality. The distribution of *pks12* SNPs is shown in A. SNPs are shown in B and cluster in lineage La1.8. The location of the SNPs in pks12 is shown in C (arrow). SNPs occur at the 3' end of *pks12*. *pks12* generally had poor mapping quality in our sample, and the SNP hotspot was always located at the end of a contig.
